# Supplementary figures and images for: Dual signaling via interferon and DNA damage response elicits entrapment by giant PML nuclear bodies
Source: eLife. 2022 Mar 23;11:e73006. doi: 10.7554/eLife.73006 (PMC8975554; doi:10.7554/eLife.73006)

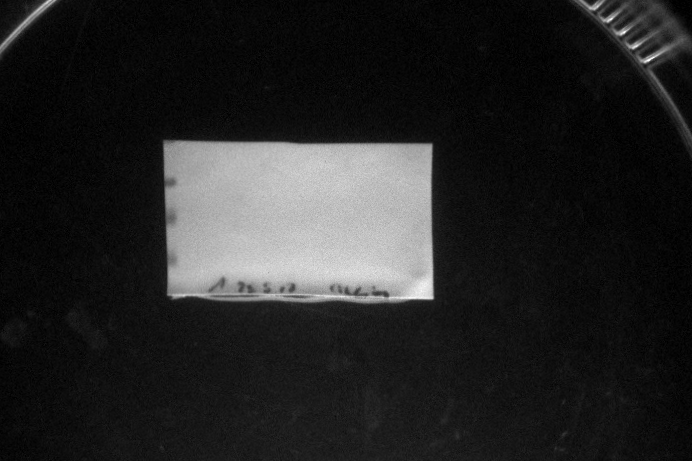

Supplement: Figure 3—source data 2. [file elife-73006-fig3-data2.zip › Figure 3d beta-actin marker.tif]

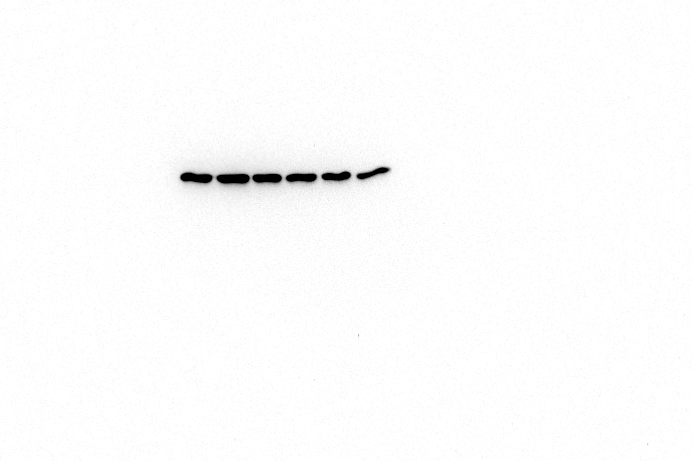

Supplement: Figure 3—source data 2. [file elife-73006-fig3-data2.zip › Figure 3d beta-actin.tif]

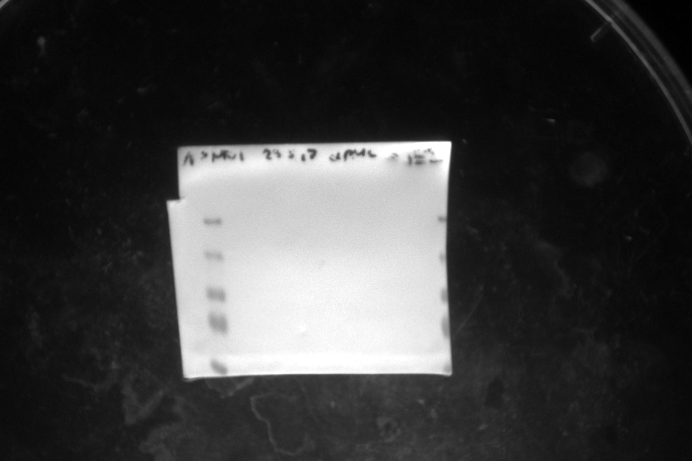

Supplement: Figure 3—source data 2. [file elife-73006-fig3-data2.zip › Figure 3d IE2 marker.tif]

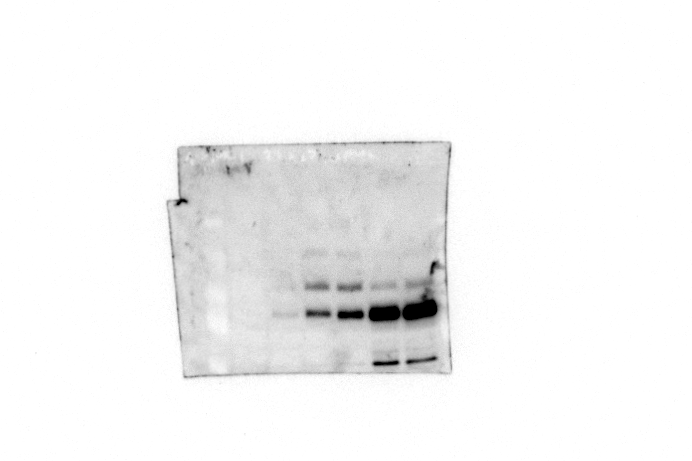

Supplement: Figure 3—source data 2. [file elife-73006-fig3-data2.zip › Figure 3d IE2.tif]

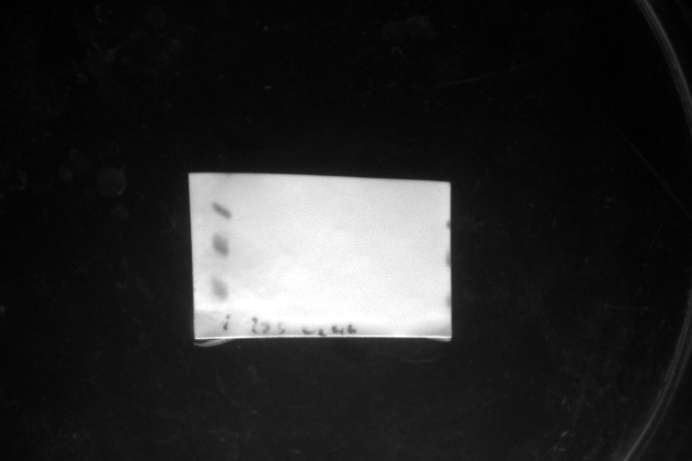

Supplement: Figure 3—source data 2. [file elife-73006-fig3-data2.zip › Figure 3d UL44 marker.tif]

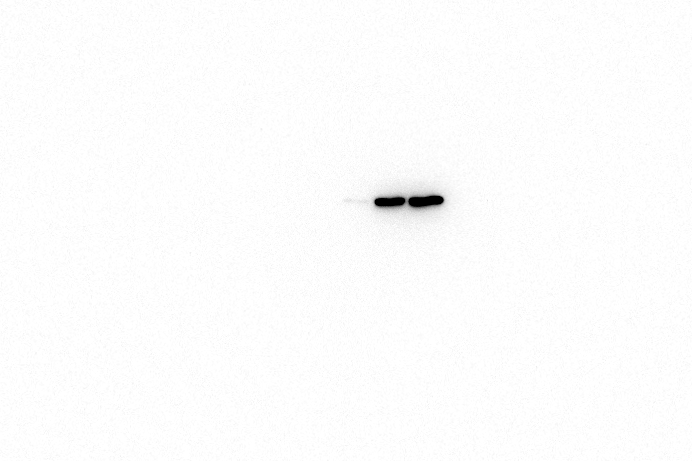

Supplement: Figure 3—source data 2. [file elife-73006-fig3-data2.zip › Figure 3d UL44.tif]

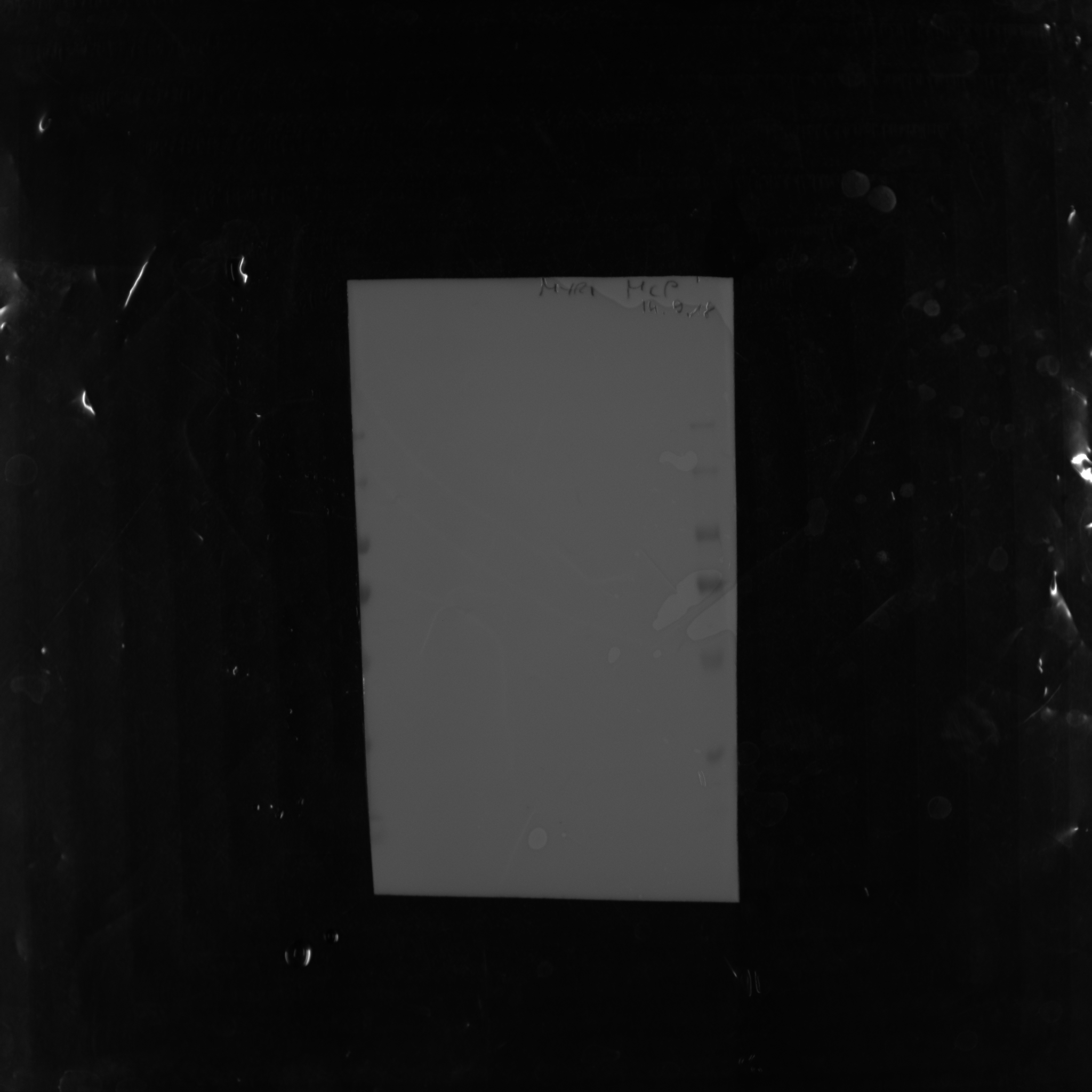

Supplement: Figure 3—source data 2. [file elife-73006-fig3-data2.zip › Figure 3d UL84 marker.Tif]

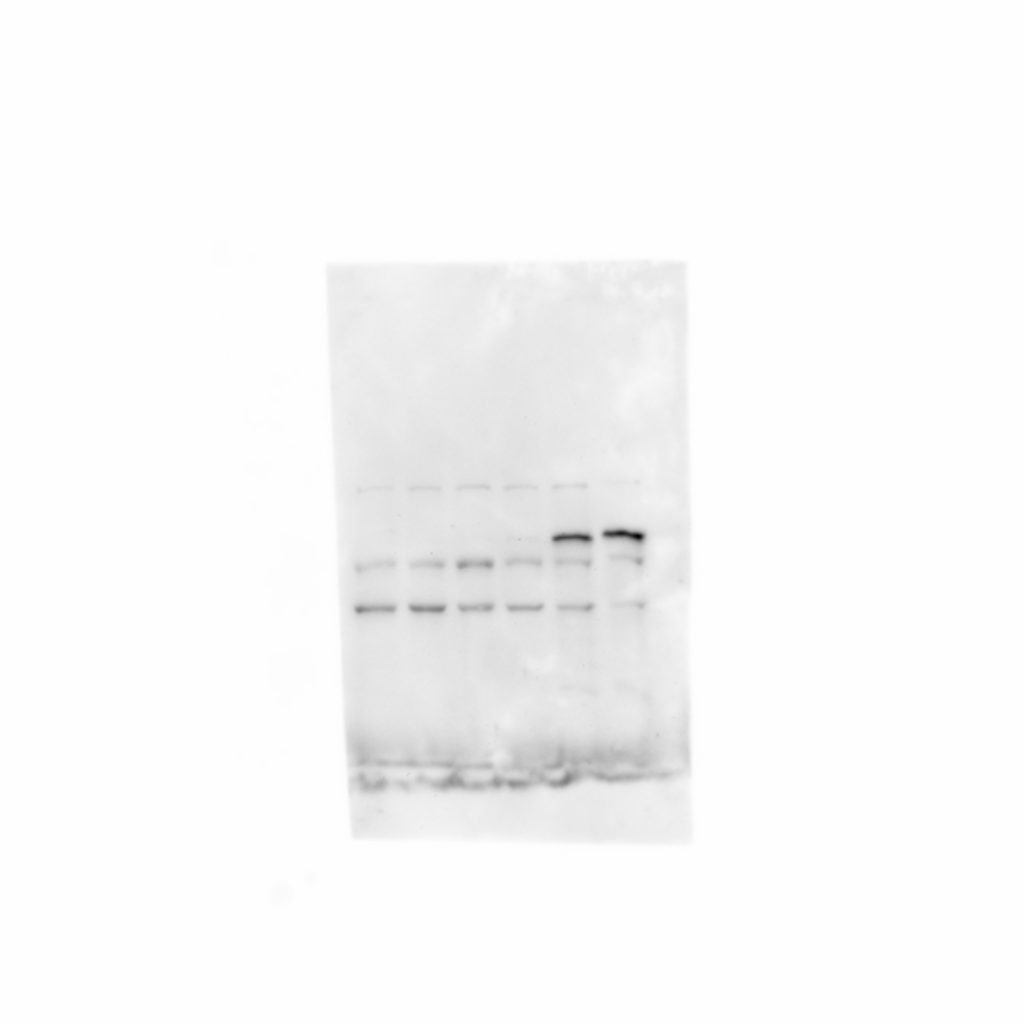

Supplement: Figure 3—source data 2. [file elife-73006-fig3-data2.zip › Figure 3d UL84.tif]

Figure 3 d

anti IE2 immunoblot

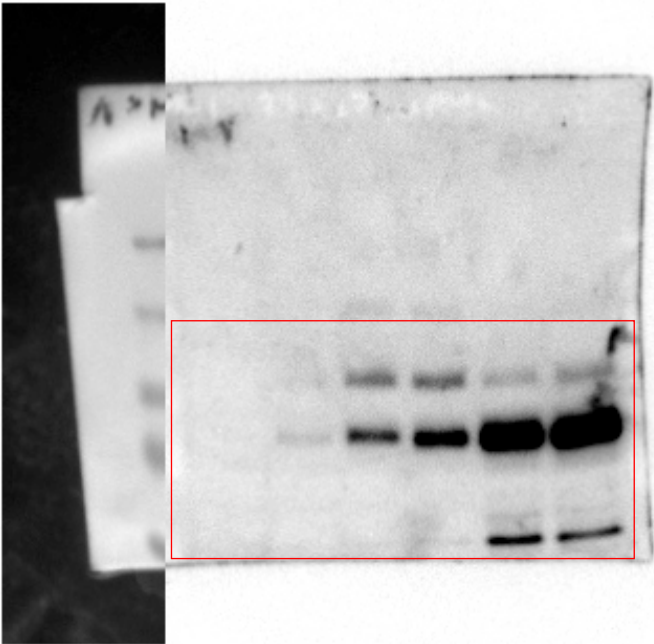

anti UL84 immunoblot

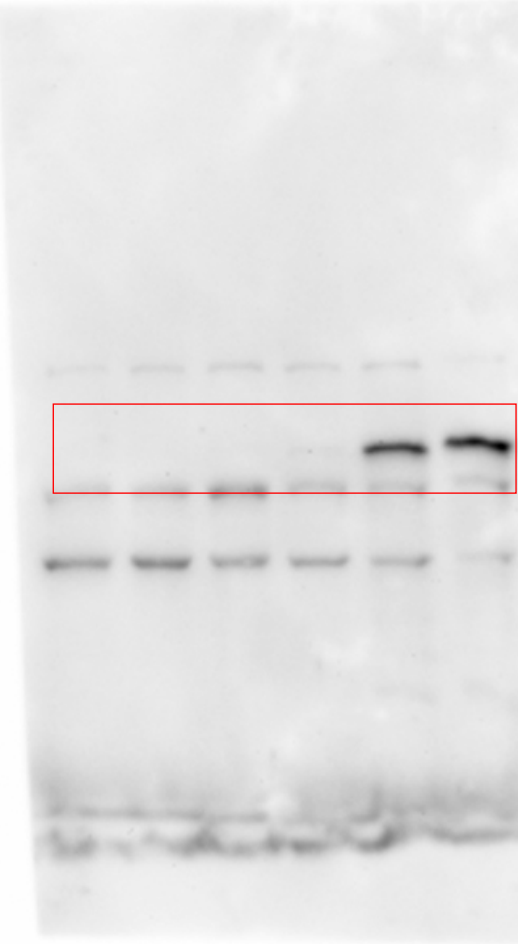

anti UL44 immunoblot

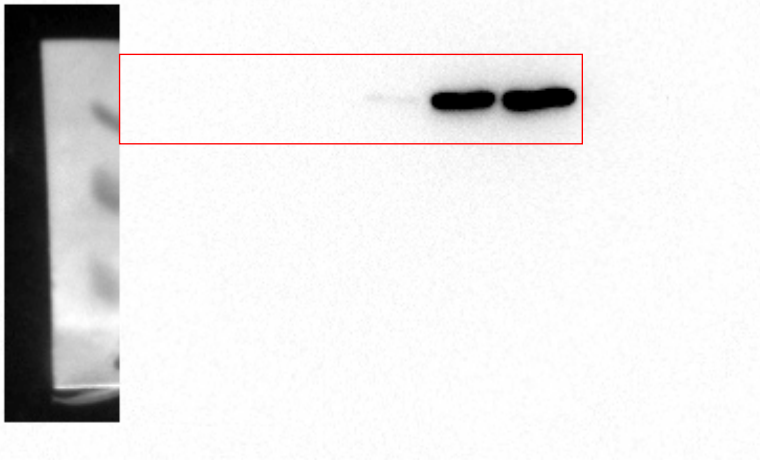

anti  $\beta$ -actin immunoblot

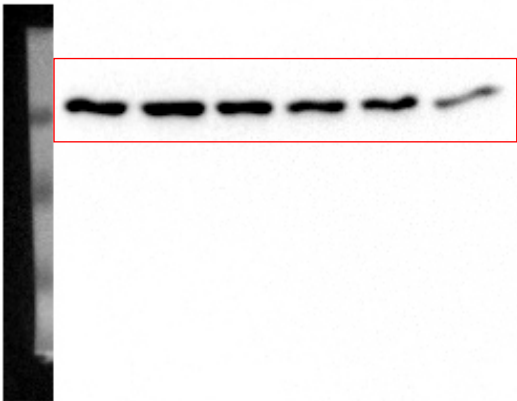

Supplement: Figure 3—source data 2. [file elife-73006-fig3-data2.zip › Figure 3d-western blots with labeled bands.pdf]

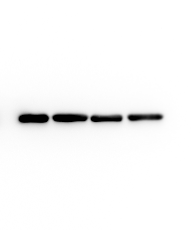

Supplement: Figure 4—source data 1. [file elife-73006-fig4-data1.zip › Figure 4a beta-actin.tif]

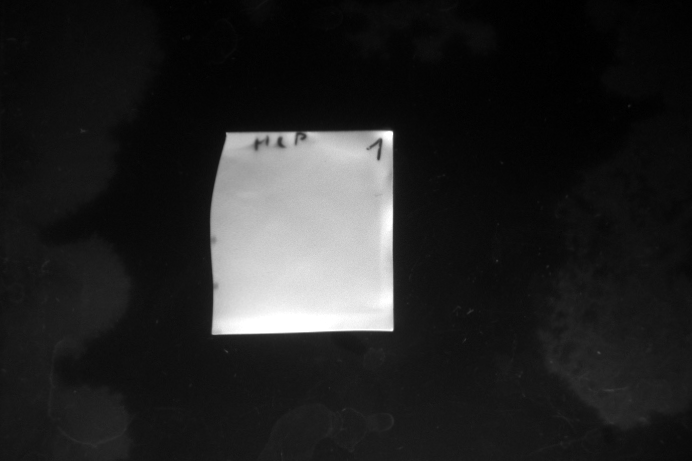

Supplement: Figure 4—source data 1. [file elife-73006-fig4-data1.zip › Figure 4a MCP marker.tif]

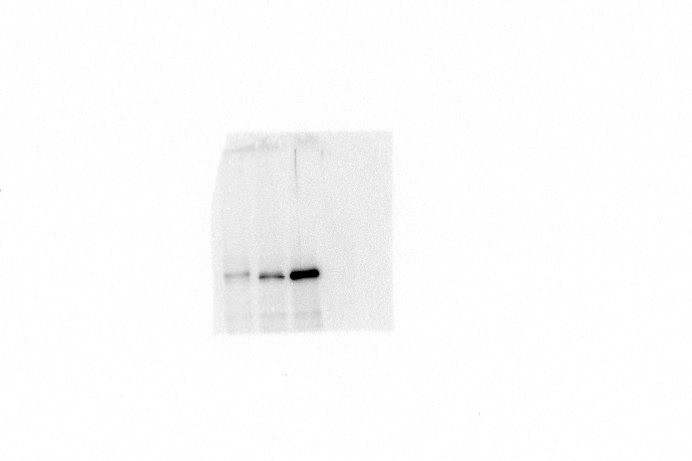

Supplement: Figure 4—source data 1. [file elife-73006-fig4-data1.zip › Figure 4a MCP.tif]

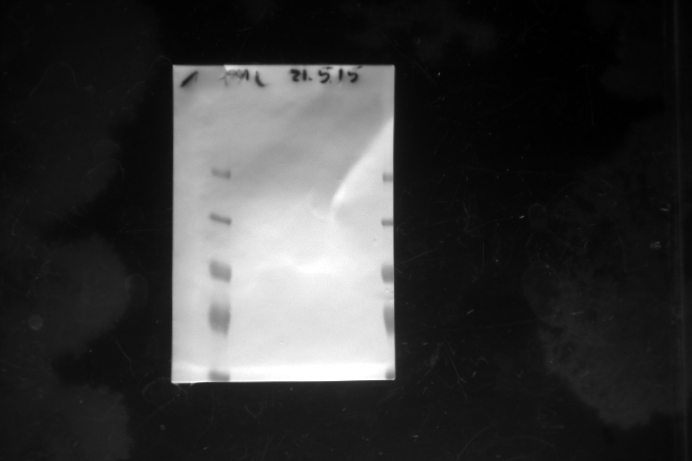

Supplement: Figure 4—source data 1. [file elife-73006-fig4-data1.zip › Figure 4a PML marker.tif]

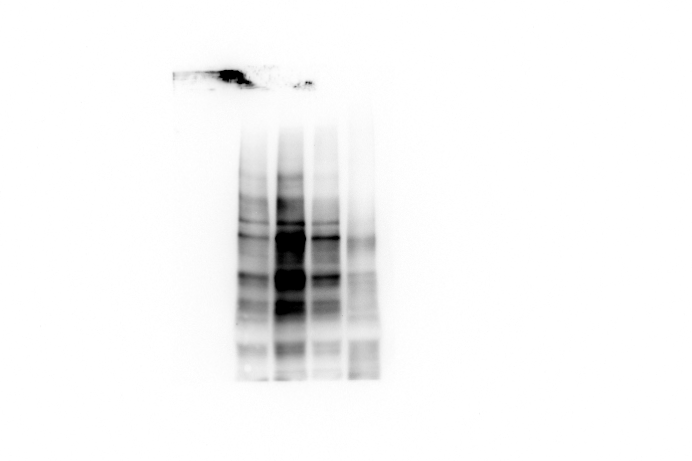

Supplement: Figure 4—source data 1. [file elife-73006-fig4-data1.zip › Figure 4a PML.tif]

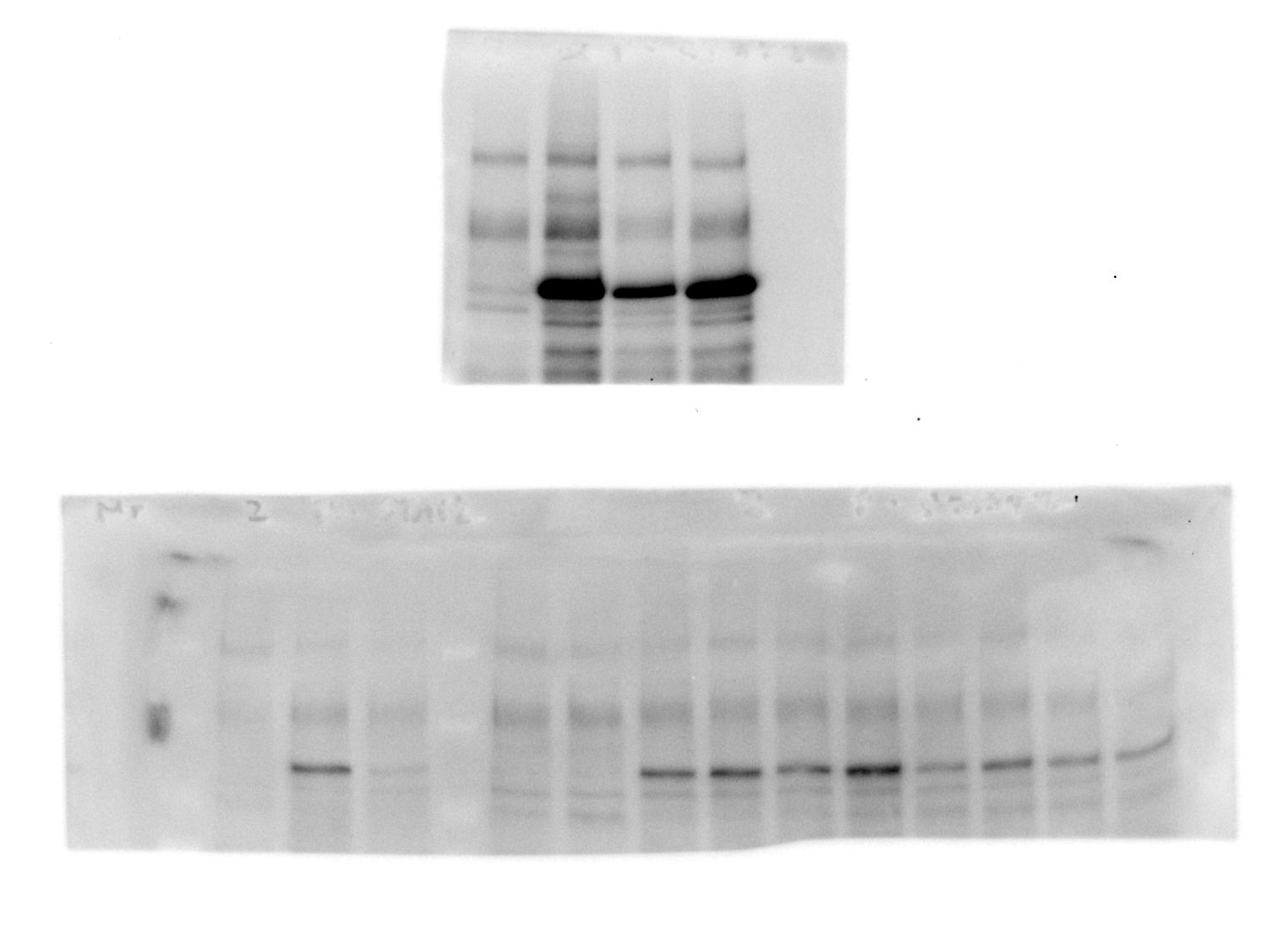

Supplement: Figure 4—source data 1. [file elife-73006-fig4-data1.zip › Figure 4a p-STAT2.tif]

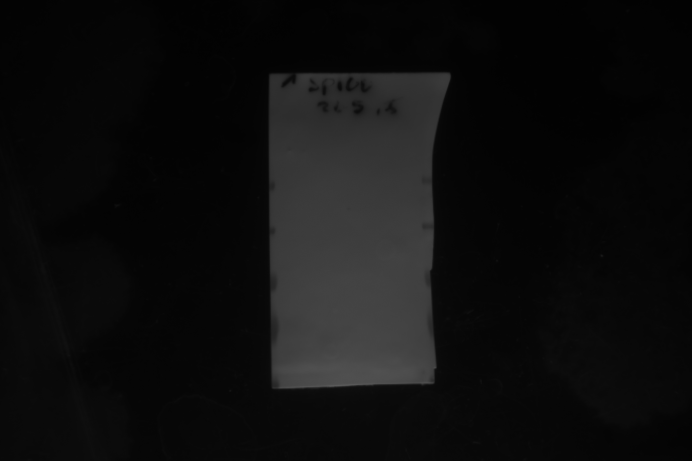

Supplement: Figure 4—source data 1. [file elife-73006-fig4-data1.zip › Figure 4a Sp100 marker.tif]

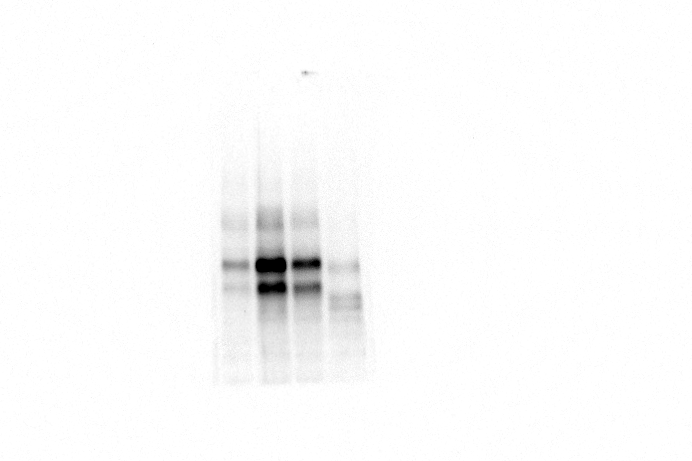

Supplement: Figure 4—source data 1. [file elife-73006-fig4-data1.zip › FIgure 4a Sp100.tif]

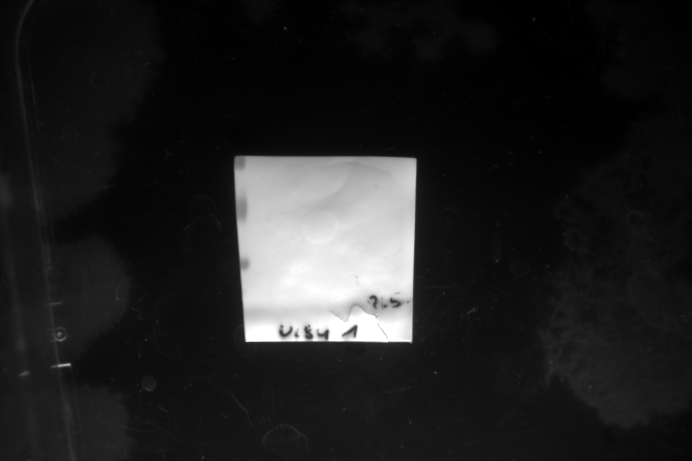

Supplement: Figure 4—source data 1. [file elife-73006-fig4-data1.zip › Figure 4a UL84 marker.tif]

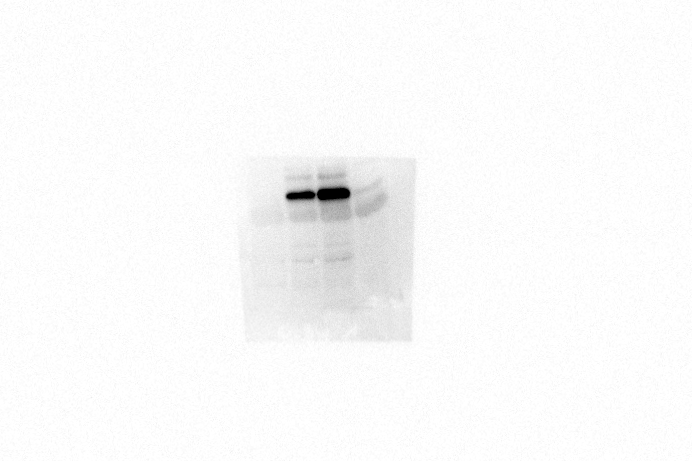

Supplement: Figure 4—source data 1. [file elife-73006-fig4-data1.zip › Figure 4a UL84.tif]

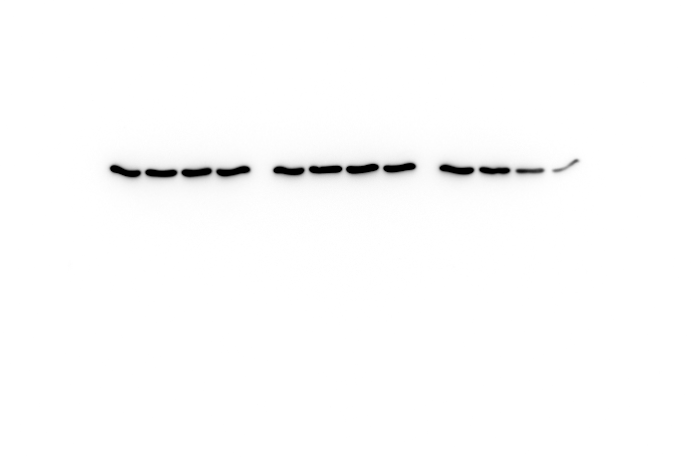

Supplement: Figure 4—source data 1. [file elife-73006-fig4-data1.zip › Figure 4d beta-actin.tif]

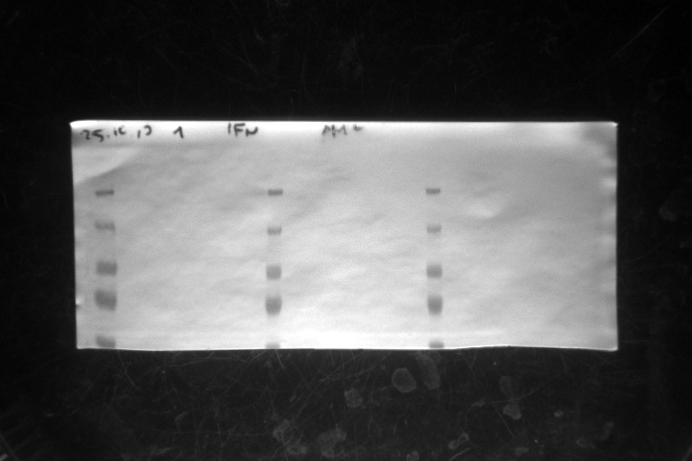

Supplement: Figure 4—source data 1. [file elife-73006-fig4-data1.zip › Figure 4d PML marker.tif]

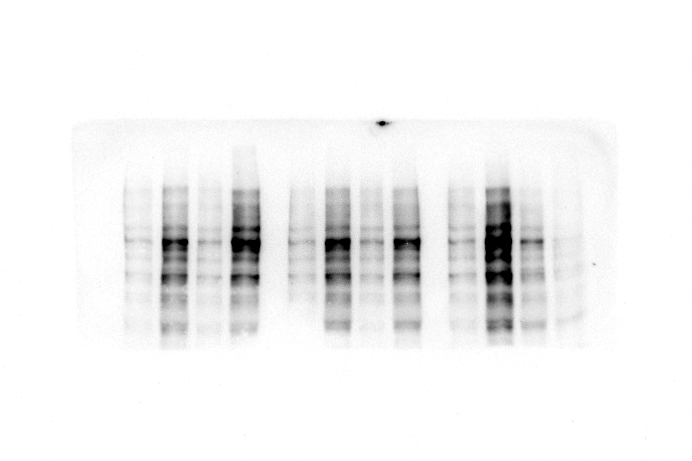

Supplement: Figure 4—source data 1. [file elife-73006-fig4-data1.zip › Figure 4d PML.tif]

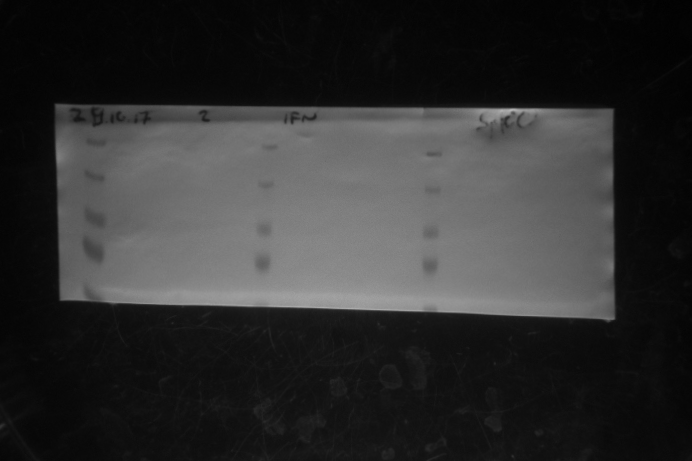

Supplement: Figure 4—source data 1. [file elife-73006-fig4-data1.zip › Figure 4d Sp100 marker.tif]

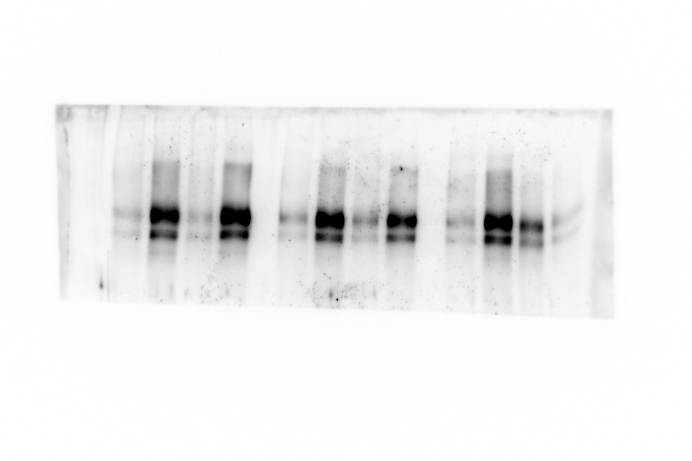

Supplement: Figure 4—source data 1. [file elife-73006-fig4-data1.zip › Figure 4d Sp100.tif]
