# Supplementary material for: Dual signaling via interferon and DNA damage response elicits entrapment by giant PML nuclear bodies
Source: eLife. 2022 Mar 23;11:e73006. doi: 10.7554/eLife.73006 (PMC8975554; doi:10.7554/eLife.73006)

Figure 4a

anti p-STAT2 immunoblot

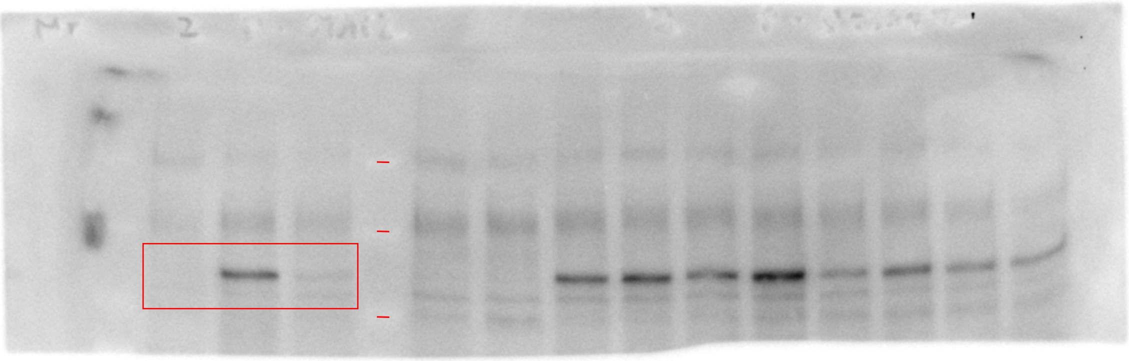

anti PML immunoblot

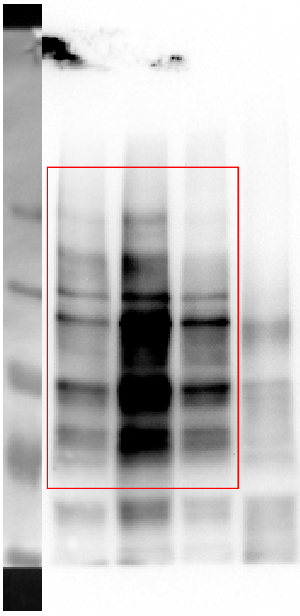

anti Sp100 immunoblot

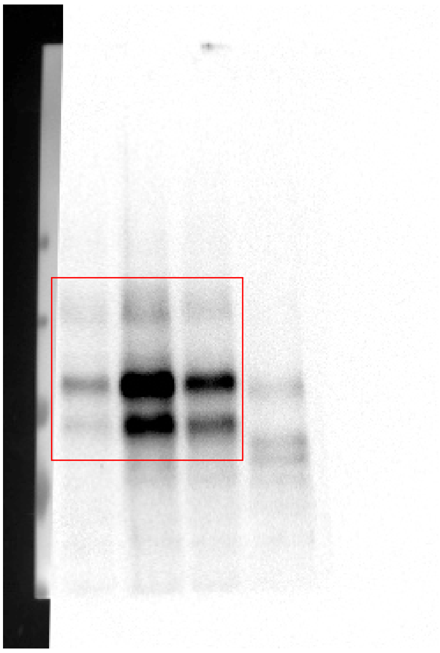

anti UL84 immunoblot

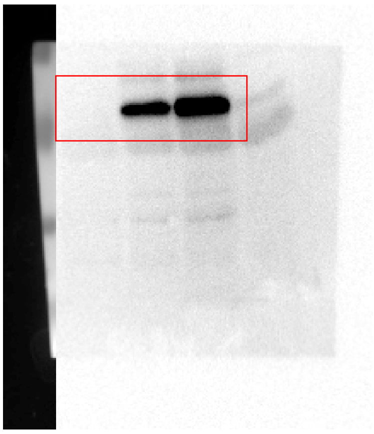

anti MCP immunoblot

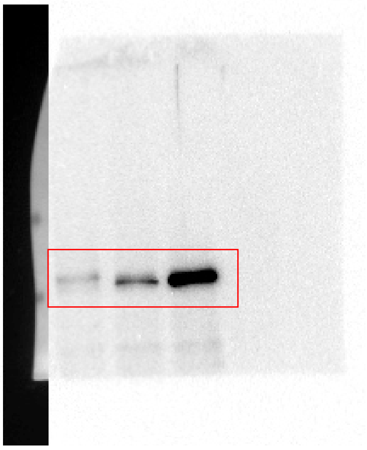

anti β-actin immunoblot

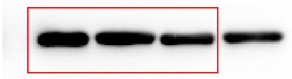

Figure 4d

anti PML immunoblot

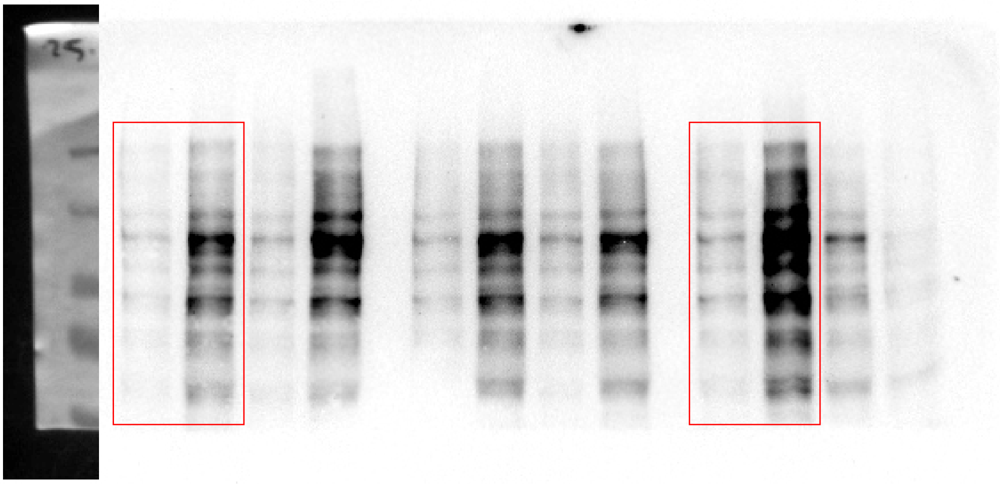

anti Sp100 immunoblot

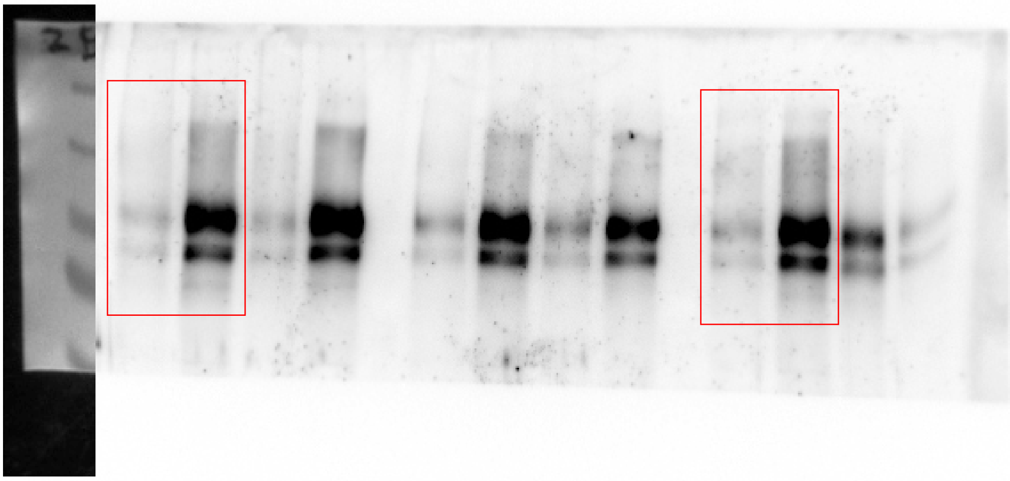

anti β-actin immunoblot

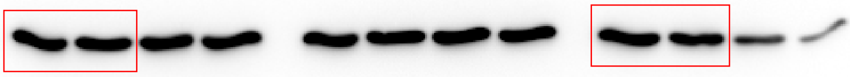

Supplement: Figure 4—source data 1. [file elife-73006-fig4-data1.zip › Figure 4a and 4d western blots with labeled bands.pdf]
