# Supplementary material for: Dual signaling via interferon and DNA damage response elicits entrapment by giant PML nuclear bodies
Source: eLife. 2022 Mar 23;11:e73006. doi: 10.7554/eLife.73006 (PMC8975554; doi:10.7554/eLife.73006)
Supplement: Supplementary file 1. [file elife-73006-supp1.docx]

| SiRNA target sequences | | |
| --- | --- | --- |
| siPML2 | AGATGCAGCTGTATCCAAG | |
| siSp100 | GGAAGCACTGTTCAGCGATGT | |
| siDaxx1 | GGAGTTGGATCTCTCAGAA | |
| siATRX | GAGGAAACCTTCAATTGTA | |
| siC | GTGCGTTGCTAGTACCAAC) | |
| Oligonucleotides for cloning of mCherry-PML into pInducer20-CRSmut | | |
| c-CRS-mut | | GCGTGTACGGTGGGAGGCCTATATAAGCAGAGCCTAGGTAGGGAGAAGTCAGATCGCCTGGAGACGCC |
| nc-CRS-mut | | GGCGTCTCCAGGCGATCTGACTTCTCCCTACCTAGGCTCTGCTTATATAGGCCTCCCACCGTACACGC |
| 5'-attB1-mCherry | | GGGGACAAGTTTGTACAAAAAAGCAGGCTATGGTGAGCAAGGGCGAGGA |
| 3' attB2-PMLVI | | GGGGACCACTTTGTACAAGAAAGCTGGGTTCACCACAACGCGTTCCTCT |
| Oligonucleotides for generation of recombinant HCMV | | |
| 5’Intron3/pKD13 | | AAAGATGTCCTGGCAGAACTCGGTAAGTCTGTTGACATGTATGTGATGTAGTGTAGGCTGGAGCTGCTTC |
| 3’Exon 4/pkd13 | | TAGTTTACTGGTCAGCCTTGCTTCTAGTCACCATAGGGTGGGTGCTCTTGATTCCGGGGATCCGTCGACC |
| IE2-eYFP-forw | | TGAGCCTGGCCATCGAGGCAGCCATCCAGGACCTGAGGAACAAGTCTCAG ATGGTGAGCAAGGGCGAGGAGCTG |
| IE2-eYFP-rev | | GGGGAATCACTATGTACAAGAGTCCATGTCTCTTTCCAGTTTTTCACTTACTTGTACAGCTCGTCCATGCCGAG |
| Primers and hydrolysis probes for real-time PCR | | |
| 5'gB_forw | | CTGCGTGATATGAACGTGAAGG |
| 3'gB_rev | | ACTGCACGTACGAGCTGTTGG |
| CMV gB FAM/TAMRA | | FAM-CGCCAGGACGCTGCTACTCACGA-TAMRA |
| 5'Alb | | GTGAACAGGCGACCATGCT |
| 3'Alb | | GCATGGAAGGTGAATGTTTCAG |
| Alb FAM/TAMRA | | FAM-TCAGCTCTGGAAGTCGATGAAACATACGTTC-TAMRA |

**Supplementary Table 1**
